# Sea Anemone Kunitz Peptide HCIQ2c1 Reduces Histamine-, Lipopolysaccharide-, and Carrageenan-Induced Inflammation via the Suppression of Pro-Inflammatory Mediators

**DOI:** 10.3390/ijms26010431

**Published:** 2025-01-06

**Authors:** Aleksandra N. Kvetkina, Anna A. Klimovich, Yulia V. Deriavko, Evgeniy A. Pislyagin, Ekaterina S. Menchinskaya, Evgenia P. Bystritskaya, Marina P. Isaeva, Ekaterina N. Lyukmanova, Zakhar O. Shenkarev, Dmitriy L. Aminin, Elena V. Leychenko

**Affiliations:** 1 Elyakov Pacific Institute of Bioorganic Chemistry, Far Eastern Branch, Russian Academy of Sciences, 690022 Vladivostok, Russia; kvetkinaan@gmail.com (A.N.K.); annaklim1991@mail.ru (A.A.K.); yliya77ya@mail.ru (Y.V.D.); pislyagin@hotmail.com (E.A.P.); ekaterinamenchinskaya@gmail.com (E.S.M.); ep.bystritskaya@yandex.ru (E.P.B.); issaeva@gmail.com (M.P.I.); daminin@piboc.dvo.ru (D.L.A.); 2 Laboratory of Structural Biology of Ion Channels, Shemyakin-Ovchinnikov Institute of Bioorganic Chemistry, Russian Academy of Sciences, 119997 Moscow, Russia; 3 Biological Department, Shenzhen MSU-BIT University, Shenzhen 518172, China; lyukmanova_ekaterina@smbu.edu.cn; 4 Laboratory of Bioengineering of Neuromodulators and Neuroreceptors, Shemyakin-Ovchinnikov Institute of Bioorganic Chemistry, Russian Academy of Sciences, 119997 Moscow, Russia; 5 Moscow Center for Advanced Studies, 123592 Moscow, Russia; 6 Interdisciplinary Scientific and Educational School of Moscow University “Molecular Technologies of the Living Systems and Synthetic Biology” Faculty of Biology, Lomonosov Moscow State University, 119234 Moscow, Russia; 7 Department of Biomedical Science and Environmental Biology, Kaohsiung Medical University, Kaohsiung 80708, Taiwan

**Keywords:** Kunitz peptide, *Heteractis magnifica*, anti-inflammatory activity, cytokines, reactive oxygen species, iNOS, carrageenan, lipopolysaccharide, histamine

## Abstract

Inflammation is a physiological response of the immune system to infectious agents or tissue injury, which involves a cascade of vascular and cellular events and the activation of biochemical pathways depending on the type of harmful agent and the stimulus generated. The Kunitz peptide HCIQ2c1 of sea anemone *Heteractis magnifica* is a strong protease inhibitor and exhibits neuroprotective and analgesic activities. In this study, we investigated the anti-inflammatory potential of HCIQ2c1 in histamine- and lipopolysaccharide (LPS)-activated RAW 264.7 macrophages as well as in LPS-induced systemic inflammation and carrageenan-induced paw edema models in CD-1 mice. We found that 10 μM HCIQ2c1 dramatically decreases histamine-induced intracellular Ca^2+^ release and LPS-induced reactive oxygen species (ROS) production in RAW 264.7 macrophages. Moreover, HCIQ2c1 significantly inhibited the production of LPS-induced tumor necrosis factor α (TNF-α), inducible NO-synthase (iNOS), and 5-lipoxygenase (5-LO) but slightly influenced the IL-1β and cyclooxygenase-2 (COX-2) expression level in macrophages. Furthermore, intravenous administration by HCIQ2c1 at 0.1 mg/kg dose reduced LPS-induced TNF-α, IL-1β, COX-2, and iNOS gene expression in CD-1 mice. The subplantar administration of HCIQ2c1 at 0.1 mg/kg dose to mice significantly reduced carrageenan-induced paw edema by a factor of two, which is comparable to the effect of diclofenac at 1 mg/kg dose. Thus, peptide HCIQ2c1 has a strong anti-inflammatory potential by the attenuation of systemic and local inflammatory effects through the inhibition of intracellular Ca^2+^ release, the production of ROS and pro-inflammatory cytokines, and enzymes involved in arachidonic acid metabolism.

## 1. Introduction

Inflammation is an innate natural process of the immune system based on the interaction of numerous different molecules such as enzymes, cytokines, chemokines, receptors, ion channels, and their ligands. When persisting for a long period, the inflammatory process can trigger various chronic diseases such as autoimmune disorders, arthritis, cardiovascular and neurodegenerative diseases, diabetes, and cancer [1]. Currently, non-steroidal anti-inflammatory drugs (NSAIDs), which inhibit cyclooxygenase (COX)-mediated prostanoid synthesis, are widely used for inflammation treatment. However, their long-term use, for example, in the cases of chronic inflammation, is limited by a number of side effects, mainly related to the risk of hospital admission for heart failure, heart attacks, and stroke [2]. Therefore, the search for new compounds with anti-inflammatory properties, offering the potential to decrease excessive inflammation associated with many diseases, is highly relevant.

Kunitz peptides were found to have great pharmacological potential as anti-inflammatory drugs. Most of them inhibit serine proteases involved in inflammation [3,4,5,6,7,8,9,10] and affect pharmacological targets such as K_V_ [3,5,11,12,13,14], Ca_V_ [15], Na_V_ [16], ASICs [17,18], and TRPV1 [19,20,21] ion channels that make these peptides prospective compounds for anti-inflammatory targeting therapy. A number of Kunitz peptides such as aprotinin, bikunin, hepatocyte growth factor activator inhibitor (HAI), and tissue factor pathway inhibitor (TFPI) inhibiting serine proteases and exhibiting different anti-inflammatory activities are actively used in the treatment of inflammatory pathologies [22].

Sea anemones were found to produce Kunitz peptides as huge combinatorial libraries (up to several dozen peptides in one species) [23,24,25], some representatives of which are being actively studied at present. We reported that HCGS1.19, HCGS1.20, and HCGS1.36 from sea anemone *Heteractis magnifica* (this sea anemone initially has been wrongly classified by us as *Heteractis crispa*) possess antihistamine activity by inhibiting histamine-induced intracellular [Ca^2+^]_i_ increase in macrophages [9,10], while K_V_ blockers and HCRG1 and HCRG2 peptides exhibit anti-inflammatory activity via a reduction in TNF-α, interleukin 6 (IL-6), and proIL-1β secretion in lipopolysaccharide (LPS)-activated macrophages [26]. In addition, HCRG1 as well as TRPV1 blocker HCRG21 [21] reduce acute local carrageenan-induced inflammation in mice model via the suppression of pro-inflammatory cytokines [14,27]. Moreover, APHC3, a TRPV1 inhibitor, alleviates inflammation-associated arthritic symptoms, such as joint swelling, pain-induced behavior, and hypersensitivity, in the CFA-induced rheumatoid monoarthritis or monosodium iodoacetate-induced osteoarthritis models and decreases IL-1β concentration in synovial fluid during prolonged treatment with APHC3 [28].

HCIQ2c1 also belongs to the Kunitz peptide combinatorial library of sea anemone *H. magnifica* [29]. Recombinant HCIQ2c1 produced in *Escherichia coli* expression system has a compact structural fold (pdb code 9IJW) stable to temperature changes up to 100 °C (58 aa, three disulfide bonds) [29,30]. We have determined that HCIQ2c1 inhibits trypsin (*Ki* 5.2 × 10^−8^ M) and forms the complexes with some inflammatory proteases such as kallikrein (*Kd* 4.90 × 10^−7^ M), cathepsin G (*Kd* 2.79 × 10^−10^ M), and human neutrophil elastase (*Kd* 1.27 × 10^−7^ M) [29]. In the in vitro models, it demonstrates neuroprotective activity against 6-hydroxydopamine (6-OHDA)- and β-amyloid-induced cytotoxicity in murine neuroblastoma Neuro-2a cells via the suppression of reactive oxygen species (ROS) production and the inhibition of ATP-induced P2X7R activation [30,31]. Moreover, HCIQ2c1 exhibits analgesic activity in various in vivo pain models as well as in the AITC-inducing inflammation model via targeting the TRPA1 channel [32]. Here, we, for the first time, studied the anti-inflammatory potential of HCIQ2c1 in the in vitro and in vivo models and identified the key mechanisms of its activity. Based on the obtained results, we consider the HCIQ2c1 peptide as a promising anti-inflammatory drug.

## 2. Results

### 2.1. Cytotoxicity of HCIQ2c1 on Murine Macrophage RAW264.7 Cells

To investigate HCIQ2c1 activity in murine macrophage RAW 264.7 cells, its cytotoxic effect on the cell line was estimated using an MTT assay. As a result, the peptide was not toxic for RAW264.7 cells at a concentration range from 0.01 to 50 μM.

### 2.2. HCIQ2c1 Regulates Histamine-Induced Calcium Release from ER in Macrophages

**Figure 1:**
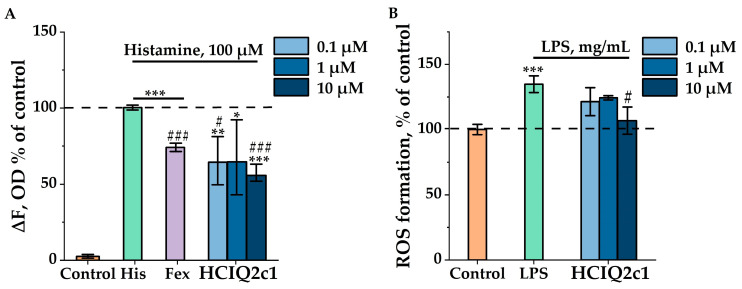
Effects of HCIQ2c1 on histamine-induced Ca^2+^ release (**A**) and lipopolysaccharide (LPS)-induced reactive oxygen species (ROS) formation (**B**) in RAW 264.7 cells. (**A**) Effects of 0.1, 1, and 10 µM HCIQ2c1 and 100 µM fexofenadine (Fex) on [Ca^2+^]_i_ induced by 100 µM histamine (His). Untreated cells were used as a control. (**B**) Effects of 0.1, 1, and 10 µM HCIQ2c1 on the ROS formation induced by 1 μg/mL LPS. Untreated cells were used as a control. The data are shown as the mean ± S.E.M. (n = 3); * *p* < 0.05, ** *p* < 0.01, and *** *p* < 0.001 indicate significant differences from the control group, while # *p* < 0.05 and ### *p* < 0.001 indicate significant differences from the His/LPS group according to one-way ANOVA/Dunnett’s multiple comparisons tests.

The ability of the HCIQ2c1 to modulate the histamine-induced Ca^2+^ release from ER was evaluated in macrophage RAW 264.7 cells loaded with the Ca^2+^-selective fluorescent probe Fluo-8AM. Notably, RAW 264.7 cells are one of the most commonly applied cells in in vitro models for the screening of anti-inflammatory and immunomodulatory compounds [33]. A strong sustained increase in [Ca^2+^]_i_ was observed after incubation of macrophages with 100 μM histamine, which was significantly blocked (to 34.8%) in the presence of 100 μM fexofenadine (Figure 1A). Similar to fexofenadine, HCIQ2c1 at the concentrations of 0.1 and 10 μM significantly inhibited histamine-evoked intracellular Ca^2+^ increase by 41.0 and 50.8%, respectively. At the same time, 1 μM HCIQ2c1 did not affect the concentration of intracellular Ca^2+^ upon histamine stimulation (Figure 1A).

### 2.3. HCIQ2c1 Regulates LPS-Induced ROS Production and Influences Pro-Inflammatory Factors Secretion

To identify the anti-inflammatory potential of HCIQ2c1 in systemic inflammation, we used the LPS-induced inflammation model in macrophage RAW 264.7 cells. LPS has been known to stimulate macrophages through TLR4, resulting in the production of ROS and the release of pro-inflammatory mediators [34]. Therefore, we evaluated the effect of HCIQ2c1 on LPS-induced ROS formation in RAW 264.7 cells by the measure of cellular oxygen bursts with the fluorescent dye DCF. The LPS stimulation of RAW 264.7 cells led to an increase in the ROS level by 34.8%. However, pre-treating the cells with 10 μM HCIQ2c1 resulted in a significant reduction in ROS production (including NO) up to the control level (Figure 1B). HCIQ2c1 at the concentration 1 μM demonstrated a lower reduction in the ROS level, but this decrease did not reach statistical significance (Figure 1B).

**Figure 2:**
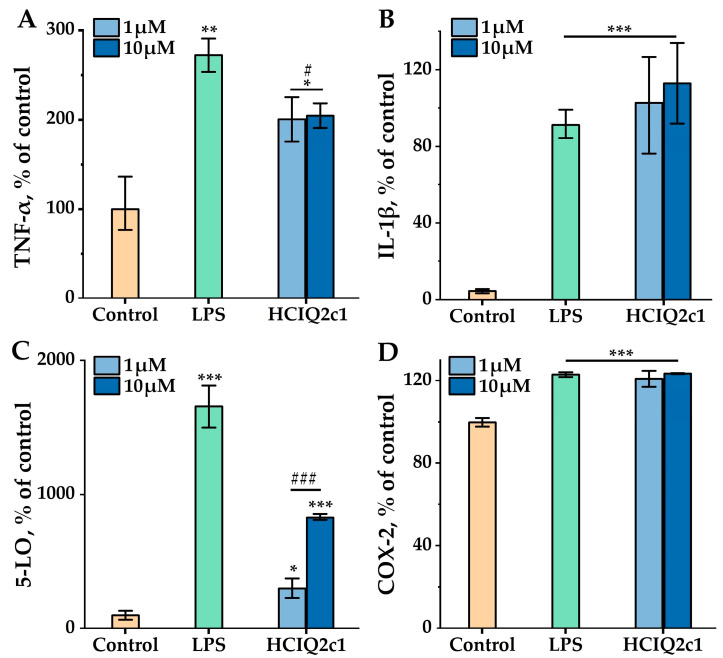
Impact of HCIQ2c1 on LPS-induced TNF-α (**A**), IL-1β (**B**) 5-LO (**C**), and COX-2 (**D**) production in RAW 264.7 cells evaluated by ELISA. Cells were pre-treated with 1 and 10 μM of HCIQ2c1 for 1 h before exposure to LPS (1 μg/mL) for 24 h. The data are shown as mean ± S.E.M. (n = 3); * *p* < 0.05, ** *p* < 0.01, and *** *p* < 0.001 indicate significant differences from the control group (untreated with LPS) and # *p* < 0.05 and ### *p* < 0.001 indicate significant differences from the LPS-treated group according to one-way ANOVA/Dunnett’s multiple comparisons tests.

LPS treatment (1 μg/mL) of RAW 264.7 cells during 24 h led to an increase in the production level of pro-inflammatory cytokines interleukin-1β (IL-1β) and tumor necrosis factor-α (TNF-α) by 86.6 and 172.5%, respectively, as well as enzymes 5-lipoxygenase (5-LO) and cyclooxygenase-2 (COX-2) by 1558.4 and 23.1%, respectively, compared to the control cells untreated by LPS (Figure 2). HCIQ2c1 at both concentrations of 1 and 10 μM significantly suppressed TNF-α production in the LPS-treated cells by 25.0 and 26.7%, respectively (Figure 2A), but no effect on IL-1β production was observed (Figure 2B). HCIQ2c1 at the concentrations of 1 and 10 μM significantly inhibited 5-LO production by 81.8 and 50.0%, respectively (Figure 2C), and did not affect the COX-2 production level (Figure 2D).

### 2.4. HCIQ2c1 Reduces Cytokine Gene Expression in LPS-Induced Inflammation in In Vivo Models

To evaluate the influence of HCIQ2c1 on cytokine production in systemic inflammation, we used an LPS-induced murine model. LPS-induced systematic inflammation was achieved by 0.1 mg/kg LPS intraperitoneal administration into CD-1 mice 60 min after the peptide or diclofenac application. To estimate TNF-α, IL-1β, COX-2, and the inducible NO-synthase (iNOS) gene expression level, quantitative PCR (qPCR) was applied and murine cDNA (first chain synthesized from total mice blood RNA) was used as a matrix.

**Figure 3:**
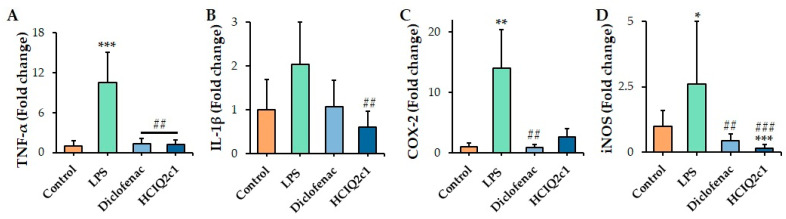
Influence of HCIQ2c1 and diclofenac on TNF-α (**A**), IL-1β (**B**), 5-LO (**C**), and COX-2 (**D**) gene expression in mice treated and untreated with LPS. HCIQ2c1 and diclofenac at the doses 0.1 mg/kg and 1 mg/kg, respectively, were administrated intravenously 30 min before the LPS (0.1 mg/kg) treatment. The results are normalized to β-actin gene expression and presented as mean ± S.E.M. (n = 7); * *p* < 0.05, ** *p* < 0.01, and *** *p* < 0.001 indicate significant differences from the control group (untreated with LPS) and ## *p* < 0.01 and ### *p* < 0.001 indicate significant differences from the LPS-treated group according to one-way ANOVA/Dunnett’s multiple comparisons tests.

LPS stimulation significantly up-regulated TNF-α, IL-1β, COX-2, and iNOS gene expression by 10, 2, 14, and 2.6 times, respectively, relative to the control mice (untreated with LPS). Enhanced TNF-α level returned to the control level under the treatment by both HCIQ2c1 and diclofenac (Figure 3A), while HCIQ2c1 almost inhibited iNOS expression (by fifteen times) and diclofenac decreased the enzyme gene expression level by six times relative to the LPS-treated group (Figure 3D). HCIQ2c1 reduced the LPS-induced IL-1β expression by three times, resulting in the return of the cytokine gene expression level to the control one; diclofenac treatment also returned the level of IL-1β to the control value, but it did not reach statistical significance. At the same time, diclofenac decreased LPS-induced COX-2 gene expression more efficiently (by fourteen times) than HCIQ2c1 (by five times). Thus, both HCIQ2c1 at the 0.1 mg/kg dose and diclofenac at the 1 mg/kg dose abolished LPS-induced increased expression of all tested pro-inflammatory factors (Figure 3).

### 2.5. HCIQ2c1 Reduces Carrageenan-Induced Inflammation

**Figure 4:**
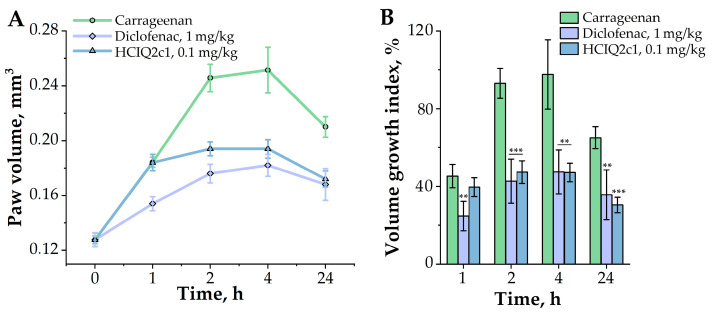
Time-dependent effects of HCIQ2c1 and diclofenac on the λ-carrageenan-induced paw volume increase (**A**) and volume growth index (%) (**B**). HCIQ2c1 (at the 0.1 mg/kg or 1 mg/kg dose) and diclofenac (at the 1 mg/kg dose) were administrated intramuscularly 60 min before the λ-carrageenan treatment. Data are presented as mean ± S.E.M. (n = 7); ** *p* ≤ 0.01, and *** *p* ≤ 0.001 indicate significant differences from the control group (did not receive HCIQ2c1 or diclofenac) according to one-way ANOVA/Dunnett’s multiple comparisons tests.

To evaluate the influence of HCIQ2c1 on local inflammation, which is characterized by swelling and redness, we used the carrageenan-induced murine paw edema model. Application of 1.5% carrageenan in a hind paw pad of mice resulted in a time-dependent increase in paw edema volume by three times compare to intact mice paw. HCIQ2c1 at a dose of 0.1 mg/kg demonstrated a 2-fold reduction in the hind paw volume after 2 h of carrageenan injection and maintained this effect for up to 4 h. The anti-inflammatory effect of HCIQ2c1 lasted more than 24 h after the carrageenan application and was comparable to the effect of diclofenac (Figure 4A). Both HCIQ2c1 and diclofenac decreased by two times the paw volume growth index 2 h after the carrageenan injection, and such an effect was observed until the end of the experiment (Figure 4B).

## 3. Discussion

**Figure 5:**
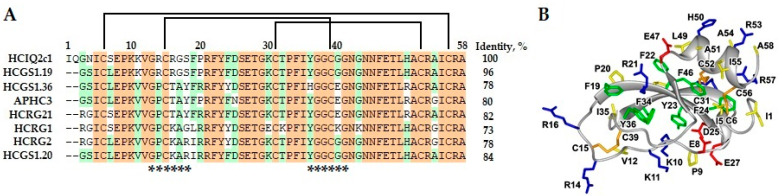
Multiple alignments of H. magnifica Kunitz peptide sequences and a 3D model of HCIQ2c1. (**A**) Multiple sequence alignment includes HCIQ2c1 (UniProtKB ID: A0A6B7FBD3), HCGS1.19 (P0DV04), HCGS1.20 (P0DV05), HCGS1.36 (P0DV06), HCRG1 (C0HJU6), HCRG2 (C0HJU7), HCRG21 (P0DL86), and APHC3 (C0HJF3). Conservative and identical residues are shown in green and orange color, respectively. The asterisks (*) below the sequences indicate the binding sites with proteases. A scheme of disulfide bonds is shown above the sequences by brackets. (**B**) The structure of HCIQ2c1 obtained by NMR (pdb code 9IJW) is shown by a gray ribbon diagram. Positively charged (+His), negatively charged, hydrophobic, and aromatic residues are colored blue, red, yellow, and green, respectively. The disulfide bonds are shown in orange.

Inspired by our previous investigations of HCIQ2c1 [29,30,32], as well as its high sequence similarity (73–96%) with its homologs from *H. magnifica* demonstrating anti-inflammatory activity (Figure 5), we proposed that similar to HCGS1.19 (the highest sequence similarity, in particular, of the protease-binding sites), HCGS1.20, and HCGS1.36 [9,10], HCIQ2c1 also possesses an anti-histamine activity. On the other hand, similarity to HCRG1, HCRG2, HCRG21, and APHC3 suggests an anti-inflammatory activity of HCIQ2c1 through the action on ion channels and subsequent suppression of pro-inflammatory cytokines [14,26,27,28]. We started with in vitro experiments to check whether HCIQ2c1 has an anti-inflammation potential and chose in vivo models to confirm this activity.

Currently, some in vitro and in vivo models are actively used for mimicking inflammation and screening bioactive compounds. Histamine, as a chemical inflammatory mediator, and bacterial lipopolysaccharide (LPS), as an inflammation trigger, are often used to stimulate inflammatory processes in different immune cells (mast cells, dendritic cells, macrophages), while carrageenan, formalin, or complete Freund’s adjuvant (CFA) are applied to induce local inflammation in rodents [35].

Histamine is an important pro-inflammatory mediator released from intracellular vesicles as a part of an immune reaction [36,37], which can lead to itching, reddening of the skin or edema, vasoconstriction of the respiratory tract, vasodilation with increased vascular permeability, or even an anaphylactic shock [38]. The allergic and inflammatory effects of histamine are mediated by the H1R activation that triggers the release of Ca^2+^ from ER via the IP3 pathway and could facilitate the movement of Ca^2+^ across the plasma membrane [36,37,39]. The dependence of the intracellular Ca^2+^ level on H1R activation was confirmed by the inhibition of all kinds of histamine-induced increases in [Ca^2+^]_i_ in human lung macrophages with the specific H1 antagonist fexofenadine [40]. In the model of histamine-activated RAW264.7 cells, we showed that HCIQ2c1 at a concentration of 10 µM reduced [Ca^2+^]_i_ increase more effectively than fexofenadine at a concentration of 100 µM (Figure 1A). Notably, peptides HCGS1.19, HCGS1.36, and HCGS1.20 demonstrated similar effects in histamine-induced murine bone-marrow-derived macrophages, but their blocking activity against [Ca^2+^]_i_ increase was comparable to fexofenadine [9,10].

On the other hand, LPS, being a component of the cellular wall of Gram-negative bacteria, plays a key role in systemic inflammation by macrophage stimulation through Toll-like receptor 4 (TLR4). As a result, the production of reactive oxygen species (ROS), pro-inflammatory cytokines (TNF-α, IL-1β), and enzymes involved in arachidonic acid metabolism (5-LO, COX-2) increases significantly in the macrophages upon the LPS treatment [33,34]. As known, a high level of ROS production results in oxidative stress, which in turn triggers signaling pathways involving the secretion of a high level of pro-inflammatory mediators and inflammation [15,16]. TNF-α is a multifunctional molecule involved in inflammation, cell proliferation and differentiation, apoptosis, and para-regulation of other pro-inflammatory cytokines [41], while IL-1β plays a key role in acute and chronic inflammatory and autoimmune disorders [42]. In most scenarios, the decreased production of both cytokines would imply a reduction in inflammation. In addition to the activation of macrophages, LPS promotes the production of 5-LO and COX-2, which are involved in leukotriene and prostaglandin synthesis, respectively, and inflammation [43,44].

In this study, we found that 10 µM HCIQ2c1 significantly reduced the ROS level to the control values (Figure 1B) and inhibited the production of TNF-α and 5-LO levels in the LPS-induced RAW264.7 cells but did not influence IL-1β and COX-2 levels (Figure 2). The inhibition of ROS formation by HCIQ2c1 is confirmed by our previous experiments on 6-OHDA cytotoxicity in murine neuroblastoma Neuro-2a cells, where HCIQ2c1 at the same concentration also significantly decreased the intracellular ROS level by 34% and increased cell viability [29]. Furthermore, COX-2 is known to be the main molecular target for NSAIDs, which inhibit both the activity and gene expression of the enzyme [45]. According to the results obtained, HCIQ2c1 appears to have an alternative anti-inflammatory mechanism differing from most NSAIDs.

Based on the data obtained about the abolishment of the histamine and LPS effects by HCIQ2c1 (Figure 1, Figure 2 and Figure 3), we can suppose that HCIQ2c1 directly targets G-protein-coupled histamine receptors and Toll-like receptors and competes with their ligands. However, HCIQ2c1 modulation of the activity of these receptors indirectly via the specific interaction with the TRPA1 channel [32] seems more feasible.

Indeed, different histamine receptors might use different downstream signaling pathways. Although exact mechanisms are still elusive, the TRP channels were found to play important roles in the sensation of histaminergic and non-histaminergic itch [46]. Recent reports have demonstrated that histamine-induced Ca^2+^ influx in the DRG neurons is mediated via H1R, H3R, and H4R and is associated with sensitivity to capsaicin, a selective TRPV1 agonist [47,48,49,50,51,52]. It was shown that histamine activates Ca^2+^ influx only in those sensory neurons that co-express H1R and TRPV1. TRPA1 is co-expressed up to 30% in a subset of the TRPV1-expressing sensory neurons [53] and can form complexes with TRPV1 [54]. In trigeminal ganglion neurons, 70% of histamine-sensitive cells respond to capsaicin and 39% to AITC, a selective TRPA agonist [55]. Moreover, the TRPA1 inhibitor HC-030031 reduces histamine-induced itch as well as itch induced by 4-MH and ST-1006, H4R agonists. The TRPV1 inhibitor SB366791, the TRPA1 inhibitor HC-030031, and the non-specific TRP channel blocker ruthenium red dose dependently reduce intracellular Ca^2+^ increase induced by histamine, 4-MH, and ST-1006 [56]. Thus, these data suggest a possible link between TRPA1, TRPV1, and HR in the histamine signal transduction.

Currently, little is known about the relationship between LPS-induced inflammation and activation of the Toll-like receptors with the TRPA1 channel. Although, recently, it was reported that sensory neurons activated by LPS demonstrated changes in extracellular and intracellular Ca^2+^ mediated by both TRPA1 and TLR4, and both receptors were involved in response to LPS [57]. It was suggested that the activation of TLR4 can result in an increased expression of TRPA1 on the cell surface, thus stimulating neuronal sensitization and pain [57].

Inspired by the results obtained in RAW 264.7 cells, we further focused on the investigation of the anti-inflammatory potential of HCIQ2c1 in two murine models of inflammation, LPS induced (in continuous of our in vitro results) and carrageenan induced, because the models are more complex and allow us to investigate systemic dependencies and indicate a larger number of inflammatory factors. According to our previous results, in the open field test, HCIQ2c1 does not exhibit neurotrophic or neurotoxic effects and has a negative influence on the locomotor system of the CD-1 mice upon intramuscular administration at doses up to 1 mg/kg [32]. Thus, the efficacy of HCIQ2c1 in the in vivo inflammation models is not a result of locomotor impairment or sedation. We found that HCIQ2c1 at a dose of 0.1 mg/kg, like diclofenac (1 mg/kg), significantly suppressed the expression level of TNF-α and IL-1β genes to the control values in the mice with systemic inflammation induced by LPS, while the decrease in COX-2 gene expression by HCIQ2c1 was less efficient than the diclofenac one (Figure 3). Moreover, HCIQ2c1 almost completely down-regulated the gene expression of iNOS, which is one of the key pro-inflammatory enzymes producing NO via the oxidation of L-arginine [58]. NO is known to play a crucial role in the pathogenesis of inflammation and has been implicated in the endotoxin-induced tissue injury modulation of cellular immunity [59].

To investigate the effects of different compounds in acute inflammation, the λ-carrageenan-induced model is widely used. λ-carrageenan is a seaweed-derived sulfated polysaccharide, the intraplanar injection of which causes an edematogenic response accompanied by inflammatory cell migration and marked nociceptive alterations [60]. The inflammatory response has been shown to be biphasic: an early phase when histamine, 5-hydroxytryptamine, and bradykinin are produced, and a delayed phase accompanied by neutrophil infiltration followed by the production of free radicals and pro-inflammatory cytokines (TNF-α, IL-1β) [61]. We showed that HCIQ2c1 decreased by two times the inflammatory paw swelling 2 h after carrageenan injection and retained this effect until the end of the experiment, which is comparable with diclofenac action (Figure 4). Notably, the anti-inflammatory effect of HCIQ2c1 was similar to the effect of Kunitz peptides HCRG1 and HCRG21 and K_V_1.3 and TRPV1 channel blockers, respectively, which, at doses of 0.1 and 1 mg/kg, reduce (up to 40%) or fully abolish the paw edema increase during 24 h after injection of carrageenan via inhibition of the TNF-α synthesis [14,62]. According to our previous electrophysiology results in *Xenopus* oocytes expressing rat the TRPA1 channel, HCIQ2c1 interacts with the open state of TRPA1 and prevents its transition to the close and inhibitor-insensitive “hyperactivated” states [32]. The modulation of TRPA activity is known to be a promising strategy for the treatment of pain and inflammation since TRPA channels, like TRPV channels, respond to inflammation being activated by pro-inflammatory cytokines [63,64]. Notably, HCIQ2c1 does not target TRPV1, but it modulates the activity of the TRPA1 channel [29,32]. Therefore, we assume that the peptide effects on histamine, carrageenan, and LPS-induced inflammation are associated either with TRPA1 regulation alone or can be associated with TRPV1 through the formation of the TRPA1/TRPV1 complexes. The modulation of the activity of this complex or TRPA1 alone, in turn, could influence the activity of histamine and Toll-like receptors through the relevant cascades; a detailed study of which we plan to conduct in the future.

## 4. Materials and Methods

### 4.1. Drugs

HCIQ2c1 was produced as described previously [29]. HCIQ2c1 stock solution was prepared in deionized water at a concentration of 10 mM, and HCIQ2c1 was diluted in PBS to the final stock concentration of 100 μM. Diclofenac and fexofenadine were purchased from Sigma-Aldrich, St. Louis, MO, USA.

### 4.2. Cell Line and Culture Conditions

The murine macrophage cell line RAW 264.7 was purchased from ATCC (TIB-71; American Type Culture Collection, Manassas, VA, USA). Cells were cultured in Dulbeccoʹs modified Eagle’s medium (DMEM) (Biolot, St. Petersburg, Russia) containing 10% fetal bovine serum (Biolot, St. Petersburg, Russia) and 1% penicillin/streptomycin (Biolot, St. Petersburg, Russia) according to ATCC’s instruction. Cells were incubated at 37 °C in a humidified atmosphere containing 5% CO_2_ (*v*/*v*). RAW 264.7 cells at a concentration of 1 × 10^4^ cells/well were dispensed into 96-well plates and incubated for 24 h in a humidified atmosphere containing 5% CO_2_ to allow cell attachment.

### 4.3. Cell Viability Assay (MTT Method)

HCIQ2c1 was added at the concentrations of 0.01, 0.1, 1.0, 10.0, and 50 μM into the cells and incubated for 24 h followed by the replacement of the medium with the tested substance in 100 μL of fresh medium. Then, 10 μL of MTT (3-(4,5-dimethylthiazol-2-yl)-2,5-diphenyltetrazolium bromide) (Sigma-Aldrich, St. Louis, MO, USA) stock solution (5 mg/mL) was added to each well, and the microplate was incubated for 4 h. After that, 100 μL of SDS-HCl solution (1 g SDS, 10 mL dH_2_O, 17 μL 6N HCl) was added to each well, followed by incubation for 18 h. The absorbance of the converted dye formazan was measured using a Multiskan FC microplate photometer (Thermo Scientific, Waltham, MA, USA) at a wavelength of 570 nm [65,66]. All experiments were repeated in triplicate. Cytotoxic activity was expressed as the percentage of cell viability.

### 4.4. [Ca^2+^]_i_ Measurement

RAW 264.7 cells, seeded at a concentration of 4 × 10^4^, were washed thoroughly using an intensive pipetting technique, placed into wells of a 96-well plate for 2 h for adhesion, and then washed with Hanks’ solution. The Fluo8/AM fluorescent calcium-sensitive probes (100 µL/well, 5 µM) (Abcam, Cambridge, UK) and 0.05% (*w*/*v*) Pluronic^®^ F-127 (Sigma-Aldrich, Burlington, MA, USA) were added to the wells in the following buffer solution: 145 mM NaCl, 10 mM glucose, 5 mM KCl, 0.8 mM MgCl_2_, 2 mM CaCl_2_, and 10 mM HEPES (pH 7.4). The plate was incubated at 37 °C for 40 min. The cellular monolayer was washed three times with the same medium, and 100 µL of the medium was added to each well followed by the incubation at room temperature for 30 min. Then, cells were pre-incubated with the examined compounds at 1 and 10 μM HCIQ2c1 or 100 µM fexofenadine for 30 min. The baseline fluorescence was measured for 2 min. Histamine solution (20 µL, final concentration of 100 µM) was added to the wells, and the change in fluorescence intensity was recorded over 1–3 min.

Fluorescence intensity was measured at λ_ex_/λ_em_ = 485 nm/520 nm using a PHERAstar FS microplate fluorescent photometer (BMG LABTECH, Ortenberg, Germany). The maximum intensity of the fluorescence of the control cells in the presence of histamine alone was taken as 100%.

### 4.5. ROS Formation Assay

RAW 264.7 cells were cultured in 96-well plates at a density of 4 × 10^4^ cells per well for 24 h. The medium was refreshed, and cells were exposed to HCIQ2c1 at concentrations of 1 and 10 µM for 60 min at 37 °C. Subsequently, cells were treated with LPS (*Escherichia coli* 055:B5, Sigma, St. Louis, MO, USA) at a final concentration of 1 µg/mL for 24 h. To assess ROS formation, a fluorescent probe, H2DCF-DA (10 µM), was added to each well, and the plate was incubated for 30 min at 37 °C as described in [67]. Fluorescence intensity was measured using a PHERAstar FS high-speed plate reader (BMG Labtech, Ortenberg, Germany) with an excitation wavelength of 485 nm and an emission wavelength of 520 nm. The data were analyzed using MARS Data Analysis software v. 3.01R2 (BMG Labtech, Ortenberg, Germany).

### 4.6. ELISA Assay

RAW 264.7 cells (2.0 × 10^4^/200 µL) were seeded in 96-well plates and incubated for 2 h at 37 °C in a 5% CO_2_ incubator for cell attachment. After that, cells were treated with HCIQ2c1 at concentrations of 1.0 and 10 µM for 1 h. Subsequently, LPS (*Escherichia coli* 055:B5, Sigma, St. Louis, MO, USA) was introduced to each well at a concentration of 1 μg/mL, and the cells were incubated for 24 h. Cells incubated without or with LPS alone were used as negative and positive controls, respectively. The samples were centrifuged at 1000× *g* for 20 min, and the resulting supernatants were collected and stored on ice for future applications. The cells were gently washed with cold PBS and then resuspended in fresh lysis buffer (0.1 mL/well). Plates containing HCIQ2c1 were subsequently centrifuged at 1500× *g* for 10 min at 2–8 °C to eliminate any cellular debris. The mixtures of supernatant and cell lysate were promptly subjected to the analysis of TNF-α, IL-1β, COX-2, and 5-LO levels using mouse TNF-α (SEA133Mu), IL-1β (SEA563Mu), COX-2 (SEB699Mu) and 5-LO (SEB355Mu) with enzyme-linked immunosorbent assay kits (Cloud-Clone Corp., Houston, TX, USA).

### 4.7. Animal Studies

The animal studies were performed under the European Commission’s legislation (Directives 86/609/EEC, 2010/63/EU), the National Standard of the Russian Federation “Good Laboratory Practice” (GOST P 53434-2009, Moscow, Russia), and the Committee on Ethics of Laboratory Animal Handling No. 05/21, 20 September 2021 protocol (PIBOC FEB RAS). Adult female CD-1 mice with weights of 25 ± 2 g were housed under a 12 h light–dark cycle at room temperature with ad libitum access to food and water. There were seven individuals in each group.

### 4.8. Lipopolysaccharide-Induced Systemic Inflammation

HCIQ2c1 at a dose of 0.1 mg/kg was injected intravenously 30 min before the test. Diclofenac (Sigma-Aldrich, St. Louis, MO, USA) at a dose of 1 mg/kg was used as a positive control and administered intravenously to animals. Control animals received an equivalent volume (30 µL) of sterile saline. Animals not receiving LPS and tested compounds were presented as intact controls. LPS (*Escherichia coli* 055:B5, Sigma, St. Louis, MO, USA) at a dose of 0.1 mg/kg was administered intraperitoneally 30 min after peptide or diclofenac injection. Ninety minutes after LPS-induced inflammation, the blood was taken from the mice for immunological analysis.

### 4.9. Carrageenan-Induced Paw Edema

HCIQ2c1 at a dose of 0.1 mg/kg and diclofenac (Sigma-Aldrich, St. Louis, MO, USA) at a dose of 1 mg/kg using a positive control were administered intramuscularly in the quadriceps muscle of the left hind paw 60 min before carrageenan injection. Animals not receiving carrageenan and tested compounds were presented as intact controls. Control animals received an equivalent volume (50 µL) of sterile saline. A total of 2 μL of 1.5% solution of λ-carrageenan was injected into the plantar skin of the hind paw pad (subplantarly) 60 min after the saline or tested compounds. The resulting volume of edema was measured using a plethysmometer (Ugo Basile, Gemonio (VA), Italy) before and 1, 2, 4, and 24 h after carrageenan injection. The anti-inflammatory activity of HCIQ2c1 or diclofenac was detected as a decrease in paw volume and the volume growth index (%) throughout the entire observation period. The volume growth index (%) was calculated using the following formula:

Volume Growth Index (%) = (Vk − Vc)/Vc × 100 (1)

where Vk is the volume of the paw after the injection of carrageenan and Vc is the volume of the paw before the carrageenan injection.

### 4.10. Quantitative Real-Time PCR

Total RNA from the whole blood cells was isolated using the Extract RNA reagent (Evrogen, Moscow, Russia) according to the manufacturer’s instructions. Briefly, the whole blood cells were homogenized in Extract RNA reagent and vortexed, and 1/5 volume of chloroform was added to the supernatant. After incubation of the mixture at room temperature for 5 min, the samples were centrifuged at 12,000 rpm for 15 min at 4 °C. The aqueous phase was transferred to a new microcentrifuge tube, and the same volume of isopropyl alcohol was added. The samples were incubated at room temperature for 10 min and were centrifuged at 12,000 rpm for 10 min. Precipitated RNA pellets were washed once with 75% ethyl alcohol and were redissolved in nuclease-free water (Ambion, Austin, TX, USA). RNA purity and quantity were evaluated using a Nanodrop One spectrophotometer (Thermo Scientific, Rockford, IL, USA).

Complementary DNA (cDNA) was synthesized from 1 μg of RNA per sample using the MMLV RT kit (Evrogen, Moscow, Russia). RNA was preliminarily treated by DNAse I (Thermo Scientific, Waltham, MA, USA). A reverse transcription reaction of 1 μg of RNA was performed in a 20 μL reaction mixture containing 4 μL of 5× MMLV RT buffer, 2 μL of dNTP mixture (10 mM), 1 μL of MMLV reverse transcriptase (100 U/μL), 2 μL DTT (20 mM), and 2 μL of oligo dT_15_-primer (20 μM) in nuclease-free water. The reaction was performed under the conditions of 37 °C for 1 h and 70 °C for 10 min using a DNA Engine amplificator (Bio-Rad, Philadelphia, PA, USA).

Quantitative PCR (qPCR) was performed using a LightCycler 96 real-time system (Roche, Basel, Switzerland), Biomaster HS-qPCR SYBR Blue (Biolabmix, Novosibirsk, Russia), and gene-specific primers for the TNF-α (NM_013693.3), IL-1β (NM_008361.4), COX2 (NM_011198.5), and iNOS (NM_001313921.1) genes. The PCR primer sequences are shown in Table 1. PCR was carried out in a 20 μL reaction mixture with 1 μL of cDNA products (1 μg/μL) as templates: 95 °C for 5 min followed by 40 cycles of denaturation at 95 °C for 10 s, annealing at 56 °C (IL-1β) or 59 °C (TNF-α), 55 °C (COX2), or 53 °C (iNOS) for 15 s and elongating at 72 °C for 25 s followed by fluorescence reading. A melting curve was drawn at the end to evaluate the specificity of PCR. The data were analyzed using LightCycler 96 Application Ver. 1.1.0.1320 (Roche, Basel, Switzerland). Quantification for each target gene expression was determined by the 2^−ΔΔCT^ method by a comparison of the experimental and control groups, where the β-actin gene was used as a reference [68].

### 4.11. Statistical Analysis

Data are presented as mean ± SEM. The number of repeats and animals (n) and statistical tests used are indicated in the figure legends. Differences in the data were considered statistically significant at *p* < 0.05. Analysis was performed using SigmaPlot 14.0 (Systat Software Inc., San Jose, CA, USA)

## 5. Conclusions

In conclusion, our study provided new insights into the anti-inflammatory activity of HCIQ2c1 belonging to the family of sea anemone *H. magnifica* Kunitz peptides discovered by us previously [29,69]. Inspired by HCIQ2c1 neuroprotective activity in vitro, which manifested through the reduction in ROS and ATP-induced intracellular Ca^2+^ release [29,30], as well as by the peptide activity as the TRPA1 modulator in the in vivo pain models [32], here, we carried out the investigation of its inflammation potential. In this study, we first showed that HCIQ2c1 is able to inhibit intracellular Ca^2+^ release in the macrophages in response to histamine, which is probably related to its ability to influence the histamine receptors via TRPA1 modulation.

On the other hand, we demonstrated that HCIQ2c1 significantly inhibits LPS-activated ROS formation, TNF-α, and 5-LO production in macrophages, which indicates the attenuation of inflammatory processes. Interestingly, the peptide had no effect on the level of LPS-induced IL-1β and COX-2, whose co-production negatively regulates 5-LO expression [70]. Nevertheless, in LPS-treated mice, HCIQ2c1 suppressed gene expression of both IL-1β and COX-2, which may be explained by the fact that the peptide can influence IL-1β and COX-2 in macrophages and mice in different ways. Notably, HCIQ2c1 almost completely suppressed LPS-induced gene expression of TNF-α and iNOS, which is an important property of anti-inflammatory drugs. Usually, the iNOS gene is expressed in conditions of inflammation, and iNOS produces large amounts of NO, which are implicated in endotoxin-induced tissue injury and the pathogenesis of inflammation [58].

Moreover, HCIQ2c1 was found to reduce the carrageenan-induced paw edema during 24 h, like the commercial drug diclofenac. The application of λ-carrageenan reproduces the acute local inflammation, symptoms of which are associated both with the production of pro-inflammatory cytokines and the activation of the TRPA1 channels [63]. Thus, HCIQ2c1, being the TRPA1 channel modulator [32], is able to prevent both scenarios. We conclude that HCIQ2c1 definitely has anti-inflammatory properties and can be considered a promising lead for anti-inflammatory therapy, and this will be tested in future controlled clinical trials.

## Figures and Tables

**Table 1:** Primer sequences used for the detection of cytokine gene expression.

Gene Names	Forward	Reverse	Annealing Temperature, °C	Fragment Length
TNF-α	5′−GTGGAACTGGCAGAAGA−3′	5′−ACTGATGAGAGGGAGGC−3′	59	192
IL-1β	5′−AACCTTTGACCTGGGCTGTC−3′	5′−AAGGTCCACGGGAAAGACAC−3′	56	144
COX2	5′−TGAGTACCGCAAACGCTTCT−3′	5′−ACGAGGTTTTTCCACCAGCA−3′	55	148
iNOS	5′−ATGTGCTGCCTCTGGTCTTGC−3′	5′−GAACCACTCGTACTTGGGATGC−3′	53	110
β-actin	5′−AGGGAAATCGTGCGTGACAT−3′	5′−AACCGCTCGTTGCCAATAGT−3′	52–60	149

## Data Availability

The additional data supporting the manuscript are available from the corresponding author upon request.
